# Application of acoustic droplet vaporization in ultrasound therapy

**DOI:** 10.1186/s40349-015-0041-8

**Published:** 2015-11-11

**Authors:** Yufeng Zhou

**Affiliations:** School of Mechanical and Aerospace Engineering, Nanyang Technological University, Singapore, 639798 Singapore

**Keywords:** Acoustic droplet vaporization, Bubble, Cavitation, Acoustic bioeffects, Ultrasound therapy

## Abstract

Microbubbles have been used widely both in the ultrasonic diagnosis to enhance the contrast of vasculature and in ultrasound therapy to increase the bioeffects induced by bubble cavitation. However, due to their large size, the lifetime of microbubbles in the circulation system is on the order of minutes, and they cannot penetrate through the endothelial gap to enter the tumor. In an acoustic field, liquefied gas nanoparticles may be able to change the state and become the gas form in a few cycles of exposure without significant heating effects. Such a phenomenon is called as acoustic droplet vaporization (ADV). This review is intended to introduce the emerging application of ADV. The physics and the theoretical model behind it are introduced for further understanding of the mechanisms. Current manufacturing approaches are provided, and their differences are compared. Based on the characteristic of phase shift, a variety of therapeutic applications have been carried out both in vitro and in vivo. The latest progress and interesting results of vessel occlusion, thermal ablation using high-intensity focused ultrasound (HIFU), localized drug delivery to the tumor and cerebral tissue through the blood-brain barrier, localized tissue erosion by histotripsy are summarized. ADV may be able to overcome some limitations of microbubble-mediated ultrasound therapy and provide a novel drug and molecular targeting carrier. More investigation will help progress this technology forward for clinical translation.

## Background

Ultrasound has been used in clinics since 1940s, mostly for diagnostic and physiotherapy purposes at low intensity. With the advance of electrics, computer technology, and transducer design in the 1980s, moderate or high-intensity ultrasound was applied in medicine with promising results. Since then, therapeutic ultrasound is emerging as an interesting and important research topic not only for physicists and engineers but also for medical practitioners. Cavitation is one of the important mechanisms in ultrasound therapy [1, 2]. However, the distribution of bubble nuclei in the soft tissue and blood is quite sparse. In order to induce the desired bioeffect in the acoustic field using low energy in vivo, artificial bubble nuclei can be introduced for cavitation. Ultrasound contrast agents (UCAs), such as gaseous microbubbles, have been used in sonographic diagnosis in cardiology clinics as cavitation enhancers in some therapeutic applications, such as blood clot fragmentation, tissue erosion, drug delivery, gene transfection, and necrosis formation in the thermal ablation [3–7]. However, UCAs have relatively large size (1–10 μm) and short in vivo circulation lifetime (i.e. a few minutes). Thus, re-administration of UCAs requires repeated sonication. Furthermore, these microbubbles cannot extravasate into tumor tissue for efficient targeting of agents to deep within the tumor.

The phenomenon of acoustic droplet vaporization (ADV), in which the phase shift of the core from liquid to gas triggered by an acoustic wave, was been described in the 1990s and was used extensively in imaging for preclinical trials [8–10]. The physics are based on the vapor pressure of the liquid, which is a function of temperature, and not necessarily based upon the liquid chemistry. In theory, ADV could be employed with any liquid that has a normal boiling point near or below the body temperature. Fluorocarbons are good candidates, particularly the perfluorocarbons (PFCs) because they have low solubility in aqueous formulations and relatively low toxicity. Perfluoropentane (PFC_5_) is perhaps the most commonly used in ADV because of its favorable transition temperature (~29 °C), its good combination of high vapor pressure, low solubility in blood, price, and availability. Due to the low solubility and diffusivity of PFC gases in water, bubbles can remain stable in an aqueous solution much longer than air bubbles in the same size [11]. Clearance of PFC nanoparticles occurs not through metabolism but slow dissolution into the surrounding medium. The resulting half-life in the tissue is much longer than that of microbubble, ranging from 4 days (i.e., perfluorooctylbromide) to 65 days (i.e., perfluorotripropylamine). Since these liquid droplets were much smaller than gas bubbles, they could traverse the lungs and provide contrast in the left heart by ADV better than the Food and Drug Administration (FDA)-approved contrast agent Albunex in the diagnosis of ventricular opacification [12, 13]. Phase-change colloids for ultrasonic imaging resulted in the advent of EchoGen™ in 1996. It shows that PFC nanodroplets (~200 nm in diameter) can be vaporized with short ultrasound pulses emitted from clinical diagnostic machines. However, the diagnostic use declined rapidly with the advent of other imaging agents. Some PFCs exhibit echogenicity due to a lower acoustic impedance than water, but not as significant as that of microbubbles. Sonography with the aid of UCAs has been proved to be a sensitive and inexpensive imaging technique in cardiovascular and oncological diagnosis. Currently, this technology has been approved by the FDA for the echocardiographic examination of wall motion abnormalities and ventricular contraction. So, the use of ADV for standard clinical imaging of cardiovascular organs and systems may not experience much resurgence in the future.

Capillaries in the diameter between 5 and 10 μm have only a single layer of endothelial cells. Although capillary vasculature in normal tissues has tight inter-endothelial junctions and therefore inhibits extravasation of nanoparticles, a variety of tumors have a porous and disorganized defective microvasculature with pore cutoff size ranging between 380 and 780 nm, which permits the so called enhanced permeability and retention (EPR) effect [14]. This characteristic allows submicron vesicles to penetrate tumor’s capillaries in a passive way. In addition to the EPR, tumors also have poor lymphatic drainage, which further ensures prolonged circulation time of nanoparticles. To avoid both extravasation to normal tissues and recognition by cells of the reticulo-endothelial system (RES), the nanoparticles are usually coated with polyethylene glycol (PEG) chains to suppress blood protein adsorption.

Microdroplets encapsulated in albumin or lipid shells in ADV are used to generate embolization and show promise for spatially and temporally targeted and substantial tissue occlusion. Both intracardiac and intravenous injections repeatedly could produce ADV in chosen arteries (i.e., renal or segmental arteries) as seen by sonography. Localized cortex occlusion was achieved with a maximum regional flow reduction of >90 % and an average organ perfusion reduction of >70 % using intracardiac injections, and vaporization from intravenous injections resulted in a substantial echogenicity increase with an average half-life of 8 min per droplet dose, which could result in the onset of cell death and possible tumor treatment via ischemic necrosis [15, 16]. Ninety percent of the pefluoropentane (PFP) concentration in the blood can be eliminated from the body within 10 min after their administration, which diminishes the risk of toxicity [17].

For nanosized droplets, surface tension effects dominate the largest expansion. Therefore, a sharp decline in the expansion factor is found in the lower nanometer range of droplets. In order to utilize the EPR effect for enhanced intratumoral diffusion, droplets of 100–200 nm in size is preferred. However, these droplets have the expansion factor of 2.5–3.5, resulting in bubbles on the order of 250–700 nm. Although the vaporized bubbles still have cavitation effects for enhanced thermal therapy or drug delivery, they are much smaller than preferable for diagnostic imaging (on the order of 1–5 μm). Post-extravasation droplet/bubble coalescence may increase echogenicity, but no in vivo evidence has confirmed such hypothesis. Therefore, there exists a design trade-off in echogenicity and droplet diffusivity [18]. In comparison, a transition occurs at the low micrometer range because of the dominant effect of the lowest ambient pressure, approximately 25 times the original diameter for frequencies between 1.5 and 8 MHz at the threshold from 4.5 to 0.75 MPa peak rarefactional pressure, respectively. This agent might be useful for tissue occlusion in cancer treatment, as well as for phase aberration corrections in acoustic imaging [8]. But, its penetration to interstitial space is limited.

**Fig. 1:**
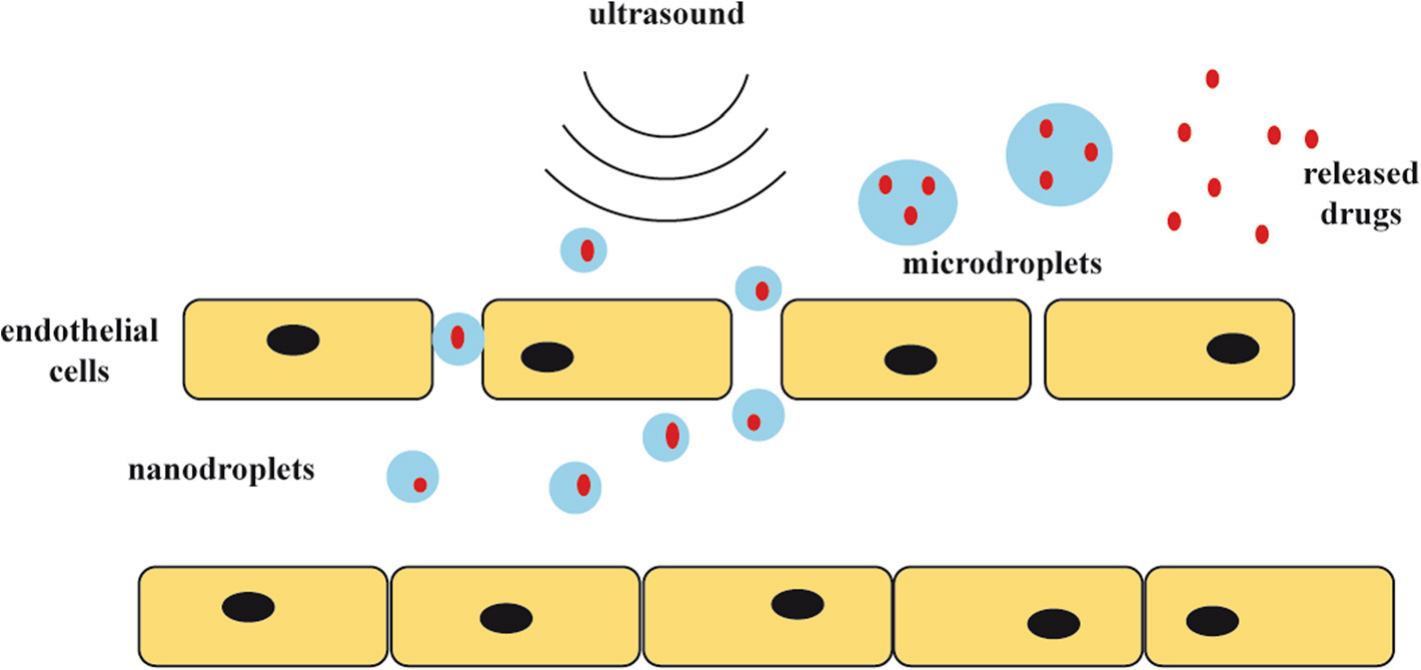
Schematic diagram of extravasation of nanoemulsion through endothelial cell gaps due to enhanced permeability and retention (EPR) and expansion to microbubbles by in situ phase transition after ultrasound exposure and release of loaded drugs

Temporally and spatially controlled therapy (i.e., drug delivery) remains a holy grail in medicine. Great effort has been devoted by generations of scientists and physicians to design an approach or system that only delivers therapy at a certain target site, leaving healthy tissue unharmed. Different internal stimuli (i.e., pH) or external stimuli, such as electromagnetic waves or magnetic and electrochemical forces, have been used to trigger such a process. Among them, ultrasound has the advantages of safety, accessibility, cost-effectiveness, and the capabilities of combined imaging and therapy. Plenty of studies are being carried out in the field of triggered drug release from microbubbles and agent uptake into cells whose membranes become temporarily permeablized due to localized acoustic bubble cavitation. So, it is reasonable to expect that phase-shift contrast agents may be a novel way of achieving ultrasound therapy with great control of spatial and temporal activation, such as localized drug delivery by nanoemulsion as shown in Fig. 1.

Acoustic activation with significant temporal and spatial specificity has led to a diverse set of in vitro and in vivo applications, not only strictly for intravascular administration because liquid PFC emulsions have a broad range of sizes. In the last decade, ADV was proposed and explored for embolic occlusion therapy, molecular imaging, drug delivery, aberration correction, high-intensity focused ultrasound (HIFU) sensitization, and tissue erosion by histotripsy [8, 16, 19, 20]. In this review paper, the physics of acoustic droplet vaporization is first introduced to understand this phenomenon. The manufacture processes are summarized briefly. Then, several medical applications are listed and followed by the discussion of the future development.

## Physics

**Fig. 2:**
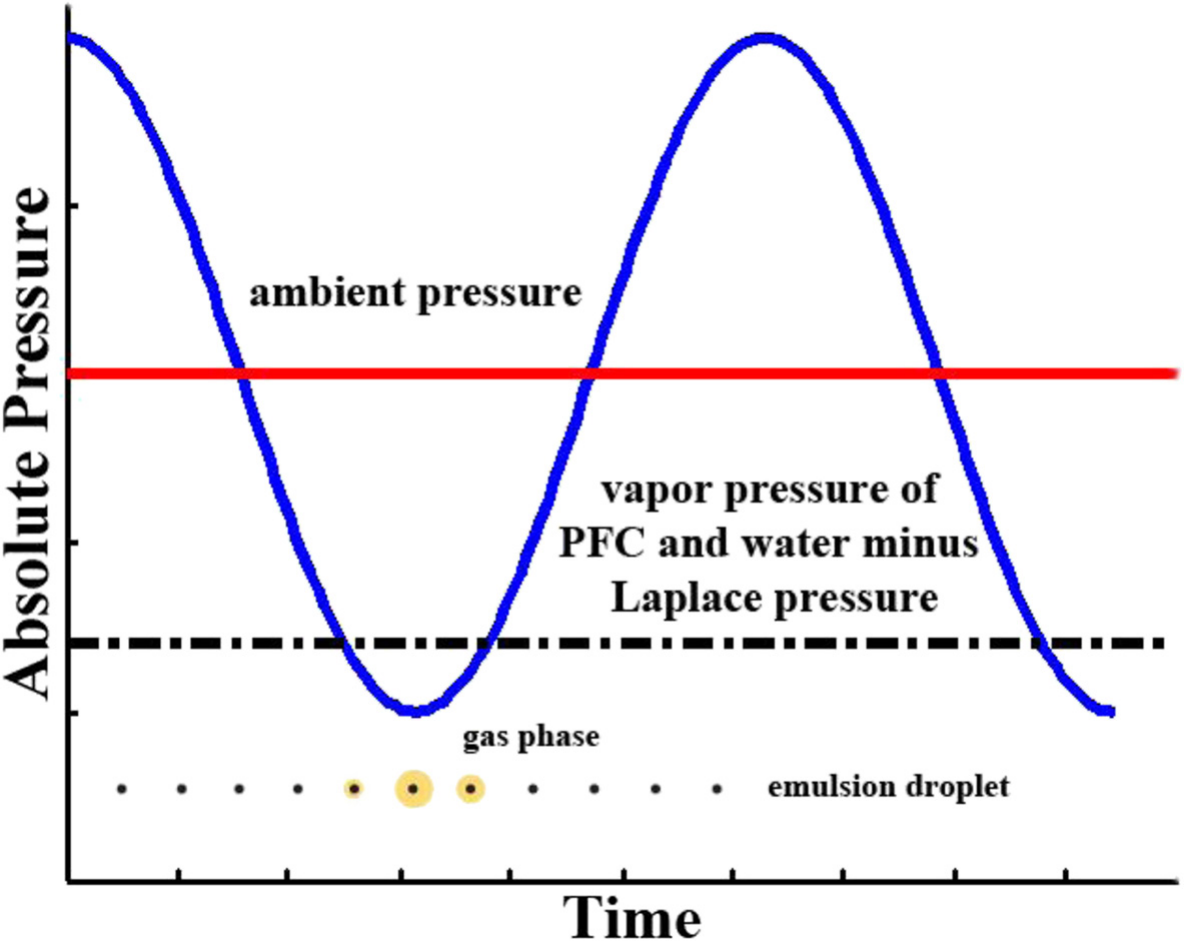
Schematic diagram of a gas phase forms around the emulsion droplet when the local wave pressure drops below the vapor pressure of the perfluorocarbon minus the Laplace pressure

Liquids possess a vapor pressure, which is defined as the pressure of the specified gas in equilibrium with its own liquid in a closed system at a specified temperature. The vapor pressure increases as the temperature of the condensed phase increases. When the surrounding pressure is greater than the vapor pressure, the condensed phase remains in its condensed form (liquid) in spite of slow dissolution and diffusion into the aqueous phase. In contrast, when the surrounding local pressure is below the vapor pressure, the liquid molecules will quickly escape to form a gas phase (boiling) without any necessary changes in temperature. The acoustic aspect of ADV occurs because the acoustic waves are used to manipulate the local pressure of the liquid, thus controlling the drive of phase transition, either from a liquid to gas or from gas to liquid. However, such transformation may not happen instantaneously because of the enthalpy required for vaporization and the presence of nucleation event required to initiate the formation of the gas phase. A liquid above its boiling point is called “superheated”. Impurities (i.e., particle nidus), foreign surfaces, and physical stresses (i.e., shear or shock event) increase the probability of nucleation within a superheated liquid. Such probability is also a strong function of the amount of superheating above the boiling point, in which superheating is often referred to as the “driving force” for nucleation. Because some PFCs have boiling points near physiological temperatures, droplets can be designed in or near a superheated state. Laplace pressure is the additional pressure imposed upon the interior fluid of a droplet because of the surface tension (or interfacial energy) between the two immiscible phases that compresses the liquid or gas inside the droplet.

1 $$ \varDelta P=\frac{2\gamma }{r(t)} $$

where *γ* is interfacial energy and *r(t)* is the bubble radius. Therefore, expansion of nanodroplets is expected to be less than that of microdroplets, and a superheated droplet can be vaporized by ultrasonic radiation into a large and stable bubble that will not return to the liquid emulsion state because of the reduction of the Laplace pressure. PFC droplets injected in vivo do not vaporize spontaneously due to the presence of Laplace pressure, and hence, their boiling point elevates [6]. The hydrophobicity of liquid PFCs leads to relatively high interfacial surface tension when dispersed in water. So, the Laplace pressure of the liquid core is on the order of atmospheres. Low boiling point of PFCs, such as perfluoropentane, enables the vaporization using lower acoustic amplitude [7]. During the phase of rarefractional acoustic pressure, the internal pressure, if less than the vapor pressure (called “subpressurization”), enables gas formation (see Fig. 2). The probability of homogeneous nucleation of a growing gas bubble is proportional to the time window (at constant subpressurization) and increases exponentially with the magnitude of subpressurization. However, subpressurization of perfluorocarbon liquid droplets can also lead to no gas formation [8, 21]. Apparent subpressurization is analogous to the apparent superheating, in which the vapor pressure at a temperature above the normal boiling point is still not greater than the local pressure inside the droplet even with sufficiently large Laplace pressure. This may be due to the absence of a nucleation event.

The ambient pressure, *P*, and the temperature, *T*, required to induce phase shift to droplet are approximately described by the Antoine vapor-pressure equation based on the derivation of the Clausius-Clapeyron relationship,

2 $$ T=\frac{B}{A-\mathit{\log}P}-C $$

where *A*, *B*, and *C* are constants determined experimentally at a particular temperature range and *P* is the ambient pressure. A modified ideal gas law equation with consideration of the Laplace pressure is used to predict the size of the expanded bubble, *r*_*g*_,

3 $$ {\rho}_lRT{r}_l^3=m{r}_g^3\left(P+\frac{2\gamma }{r_g}\right) $$

where *r*_*l*_ is the radius of the liquid droplet, *m* is the molar mass, *R* is the ideal gas constant.

**Fig. 3:**
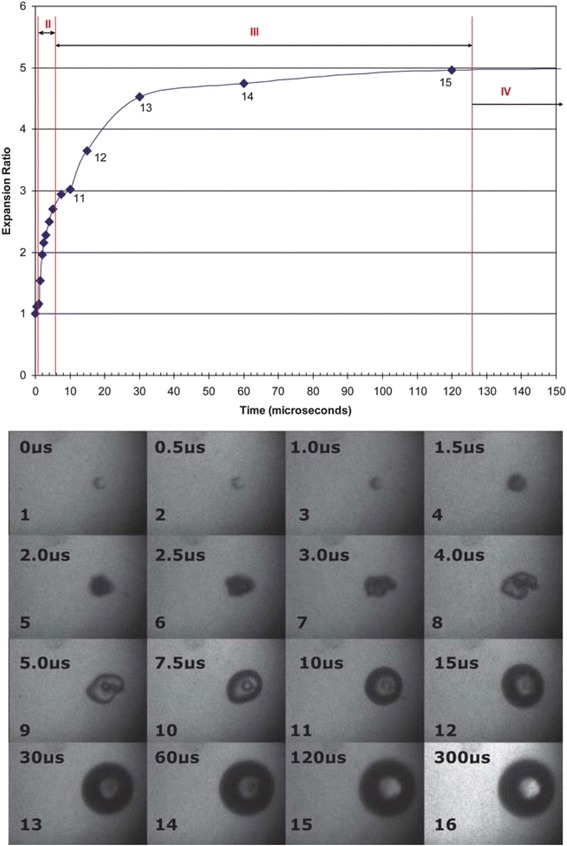
High-speed photography and size of ADV process with initial droplet diameter of 17 μm produced by a single 17-cycle ultrasonic pulse at a frequency of 3.5 MHz and peak negative pressure of 10.8 MPa, with courtesy of [22]

ADV bubble dynamics can be described by multiple stages following empirical laws (see Fig. 3) [22]: (1) a microsecond-long nucleation stage with a linear growth rate; (2) a stage lasting several microseconds, where a linear growth rate is observed, but smaller than the previous stage; and (3) a stage up to 200 μs in duration with a nonlinear growth rate where the vapor bubble reaches 90–95 % of its final size, before asymptotically reaching its final size. PFC mass evaporation rate may be predicted by the kinetic theory limits to the mass flux that can be attained in the phase change process [23] and validated in experiment on a time scale up to 600 μs [22].

**Fig. 4:**
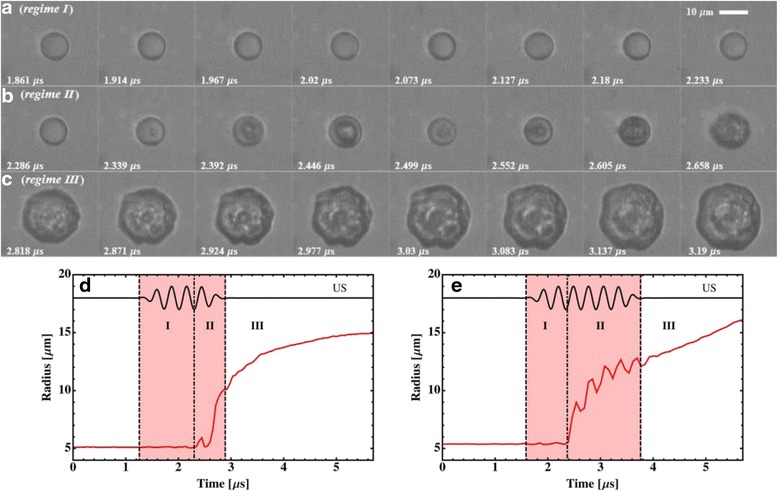
**a**–**c** A set of consecutive images showing acoustic droplet vaporization taken at 18.5 Mfps at an ambient temperature of 41 °C in three distinct regimes by 10-cycle ultrasonic pulse at a frequency of 3.5 MHz and peak negative pressure of 4.5 MPa and **d**, **e** two typical examples of the time dependent curves of the outer radius of the droplet/bubble complex obtained from the vaporization at ambient temperature of 41 and 50 °C, respectively, with courtesy of [24]

The droplet vaporization dynamics have three distinct regimes (see Fig. 4). Regime I begins with the start of ultrasound and is prior to nucleation. It is characterized by shape deformations and oscillatory translations of the droplet within the surrounding fluid on the order of 100 nm along the propagation direction of the applied ultrasound. During the oscillatory translations, the droplets experience a random walk component. Regime II is characterized by the growth of a vapor bubble during the ultrasonic exposure enhanced by ultrasound-driven rectified heat transfer. The growth of the vapor bubble in this regime is composed of two components at different time scales. The first component is a monotonic expansion with a typical velocity around 5–10 m/s at a typical size near 10 μm. The second component is a sinusoidal perturbation due to successive vaporization and condensation cycles driven by the applied ultrasound. Nucleation is a highly stochastic process and could occur as early as in the second pressure cycle. The high phase conversion rate and the rapid bubble growth in this regime suggest the dominant role of rectified heat transfer. Rectified heat transfer is the net effect of the decreased heat transfer during the contraction of the vapor bubble surface, which is lower than the increase of heat transfer during the surface expansion. Regime III is a continued growth of the vapor bubble fully dominated by heat transfer after the termination of ultrasound exposure and is characterized by a monotonic and relatively slow expansion. The overpressure between the inside and outside of the vapor bubble in regime III is small (of order 5 kPa). It suggests that the inertial processes occur fast enough to allow the vapor bubble growth and are not limiting this process [24].

Acoustic cavitation is one possible mechanism of vaporization. However, the ADV threshold is found less than the inertial cavitation (IC) threshold. Thus, an IC event is not necessary to cause vaporization, though IC nuclei, such as ultrasound contrast agent and polystyrene microspheres, external to the droplet can lower the ADV threshold. ADV occurs first and may provide a bubble then going through IC. Because the IC threshold remained constant with different gas saturation of the bulk fluid and the droplet diameter, it is likely that the nucleus for IC is not external to the droplet and may be the ADV bubble itself. In addition, it is possible to achieve ADV with or without IC [25]. Meanwhile, the intensity required to produce higher harmonics (the onset of stable cavitation) using the Fourier transform to the acoustic emission signals measured by passive cavitation detection (PCD) was about 10-fold greater than the occurrence of the fundamental frequency (phase shift). The acoustic intensity required for the baseline shift (the onset of IC) was about sixfold higher than that required for the higher harmonic frequencies [26]. Larger droplet requires less acoustic intensity to produce IC. PFC_6_ nanoemulsions at 476 kHz required higher intensity for phase shift compared to PFC_5_ while opposite conclusions were found at 20 kHz [26].

**Fig. 5:**
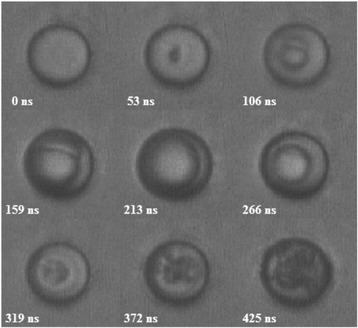
Consecutive images of ADV at 15 °C superheat produced by ultrasound at frequency of 3.5 MHz and pressure of 4.5 MPa with the initial droplet radius of 5.4 μm, with courtesy of [76]

High-speed photography of the nucleation of the gas phase shows that the bubble forms within the liquid PFC and not at the liquid PFC/water interface [27–29]. The ADV threshold has been found to decrease with increasing frequency, whereas the cavitation threshold in liquids is expected to increase with increasing frequency. The threshold pressure decreases with increasing size of the droplet. Acoustic droplet vaporization is initiated by a combination of two phenomena: highly nonlinear distortion of the acoustic wave before it hits the droplet and focusing of the distorted wave by the droplet itself. At high excitation pressures, nonlinear distortion causes significant superharmonics with wavelengths on the order of the droplet size (see Fig. 5). These superharmonics strongly contribute to the focusing effect. The focused pressure increases the chance of nucleation. The larger the droplet size, the stronger the focusing. The smaller nanodroplets will require a higher frequency for activation [30]. Smaller droplets (<20 μm) had the initial nucleation site in the hemisphere closer to the ultrasound source versus initial nucleation in larger droplets (>20 μm) formed further from the ultrasound transducer. As droplet size decreased, the wavelength begins to be of similar or longer than the droplet diameter, and the initial nucleus traverses across the axis of acoustic propagation and forms proximal to the acoustic source. For droplets smaller than the half wavelength, the carrier frequency is unable to refocus in the droplet; thus, inclusion of harmonics better describes the location of the initial nucleation for small droplets. This may suggest an increased reliance on higher pressures from higher harmonics for ADV of smaller droplets. Mechanistically, acoustic lensing within the droplet may enhance the development of large local negative pressure resulting in a cavitation-like event (i.e., nucleation site formation and onset of the ADV process). Once formed, the nucleation site appears stable and serves as a source for conversion from liquid to gaseous PFC, generating a high-pressure bubble quickly undergoing rapid expansion to its equilibrium diameter [31]. The extent of superharmonic focusing is negligible for the droplet size (<400 nm) and frequencies (≤3 MHz).

The behavior of vaporized submicron droplets immediately following vaporization is different to their micronsized counterparts. The efficiency of droplet vaporization is dependent on the initial droplet nucleation rate, and the stability of the newly created bubbles following vaporization is determined by recondensation into the liquid form due to an increase in Laplace pressure. There is a threshold bubble radius of approximately 800 nm (in concordance with the theoretical prediction using the Homogeneous Nucleation Theory) above which the bubbles are stable against recondensation and below which uncoated bubbles are prone to recondensation. Long interval time does not significantly affect the threshold pressure for recondensation. The efficiency of initial droplet nucleation is quite high (on the order of at least 10 %), as approximately ten droplets may be sufficient for the production of a detectable amount of bubbles with comparable echogenicity as the single microbubble in sonography. However, up to 90 % of the newly formed bubbles may recondense into the liquid, resulting in the efficiency of stable bubble generation dropping to even below 1 %. Following vaporization and bubble formation, the newly created bubbles retain their initial coating material and undergo rapid expansion (up to 10 μm in diameter) followed by stable oscillations for a number of cycles eventually coming to rest or rapid shrinkage or collapse to a radius of less than 1 μm. During the initial growth and collapse, the bubbles may undergo fragmentation and coalescence. There is no dependence of bubble coalescence rate on ultrasound excitation pressure. The probability of bubble survival is significantly higher for coalesced bubbles than the noncoalesced ones following vaporization because of increased total volume and size of the bubble as well as the reduced Laplace pressure, but decreased with the excitation acoustic pressure. Presence of a shell reduces the effective surface tension on the bubble and subsequently the Laplace pressure. Bubbles with higher concentration of surfactant coating are more likely to avoid condensation than those with less surfactant. Coalescence of bubbles may also increase the surfactant concentration on the final combined bubble. Uncoated PFP bubbles above the radius of approximately 1 μm do not recondense but dissolve on the order of milliseconds [32].

Bubble expansion may not follow the prediction of the ideal gas law because of the influx of dissolved gases from the surrounding medium. The influx of dissolved oxygen into a gas phase inside the emulsion occurs during the rarefactional phase of the acoustic wave, followed by the rectified diffusion of dissolved gases into the formed bubble. The solubility of gas increases with pressure according to Henry’s law. Although some gases may be released during the acoustic compression phase, its degree is less than the uptake due to higher gas solubility under pressure and a smaller surface area of the compressed bubbles. When the acoustic wave is terminated, equilibrium corresponding to the ambient pressure is restored; gases with super-equilibrium concentrations diffuse out, thus restoring nanodroplets. The noncondensable gas will not condense along with the PFC and may not completely dissolve into the surrounding liquid, leaving a very small bubble of noncondensable gas that easily nucleates in the next cycle of PFC boiling, leading to an even larger bubble, and subsequently more diffusion of noncondensable gas into the bubble. The vapor pressure of water is 3.17 kPa at 25 °C, an order lower than that of a small PFC (i.e., 29.1 kPa for PFC_6_). Thus, PFC bubbles will form before homogeneous nucleation of water vapor bubbles. However, in practice, a small portion of water evaporates into the first bubbles to form. The first perfluoropentane bubble to form contains 3.5 and 4.4 % water vapor in equilibrium at 25 and 37 °C, respectively. Perfluorohexane has a lower vapor pressure, so the corresponding values are slightly higher, 9.8 and 11.6 % at 25 and 37 °C, respectively [33]. The complexity in the vapor components would lead to complicated bubble dynamics after vaporization.

Till now, some work on the theoretical model of ADV has been carried out. The bubble dynamics with a phase shift can be described by Pitt modification of the Raleigh-Plesset equation [33]

4 $$ \frac{P_g\left(T,t\right)-{P}_{\infty }(t)}{\rho_L}=R\frac{d^2R}{d{t}^2}+\frac{3}{2}{\left(\frac{dR}{dt}\right)}^2+\frac{4{\mu}_L}{R}\frac{dR}{dt}+\frac{2\gamma }{R{\rho}_L} $$

where *μ*_*L*_ is the kinematic viscosity of the water phase,

5 $$ \begin{array}{c}\hfill {P}_{\infty }={P}_{atm}+{\rho}_wgh+A\mathit{\sin}\omega t\hfill \\ {}\hfill {P}_g={P}_{vap,PFC}\left({T}_{s,PFC}\right)+{P}_{vap,{H}_2O}\left({T}_{s,{H}_2O}\right)\hfill \\ {}\hfill {P}_0={P}_g-\varDelta P\hfill \end{array} $$

The vapor pressure of PFCs and water can be obtained from empirical equations in the form of

6 $$ {P}_{\mathrm{vap}}\left({T}_s\right)= \exp \left(A+B/{T}_s+C \ln {T}_s+D{T}_s^E\right) $$

where *A*, *B*, *C*, *D*, and *E* are constants. This partial differential equation (PDE) is solved by the explicit Runga-Kutta routine that uses a fourth order approximation to calculate and a fifth order to truncate the solution. The maximum bubble size and collapse velocity are found to increase with ultrasound amplitude, droplet size, vapor pressure, and temperature for sufficient time. Sufficient time means the duration in which the local pressure is lower than the vapor pressure needed to produce heterogeneous or homogeneous nucleation. The liquid surface cools in the liquid evaporation phase while it heats up during the condensation of gas molecules. Theoretical simulation shows that the radius of the bubble and the bubble collapse velocity increases almost linearly with acoustic amplitude. The bubble collapse velocity is also fairly sensitive to ultrasound frequency, temperature, the surface tension, as well as initial droplet size.

**Fig. 6:**
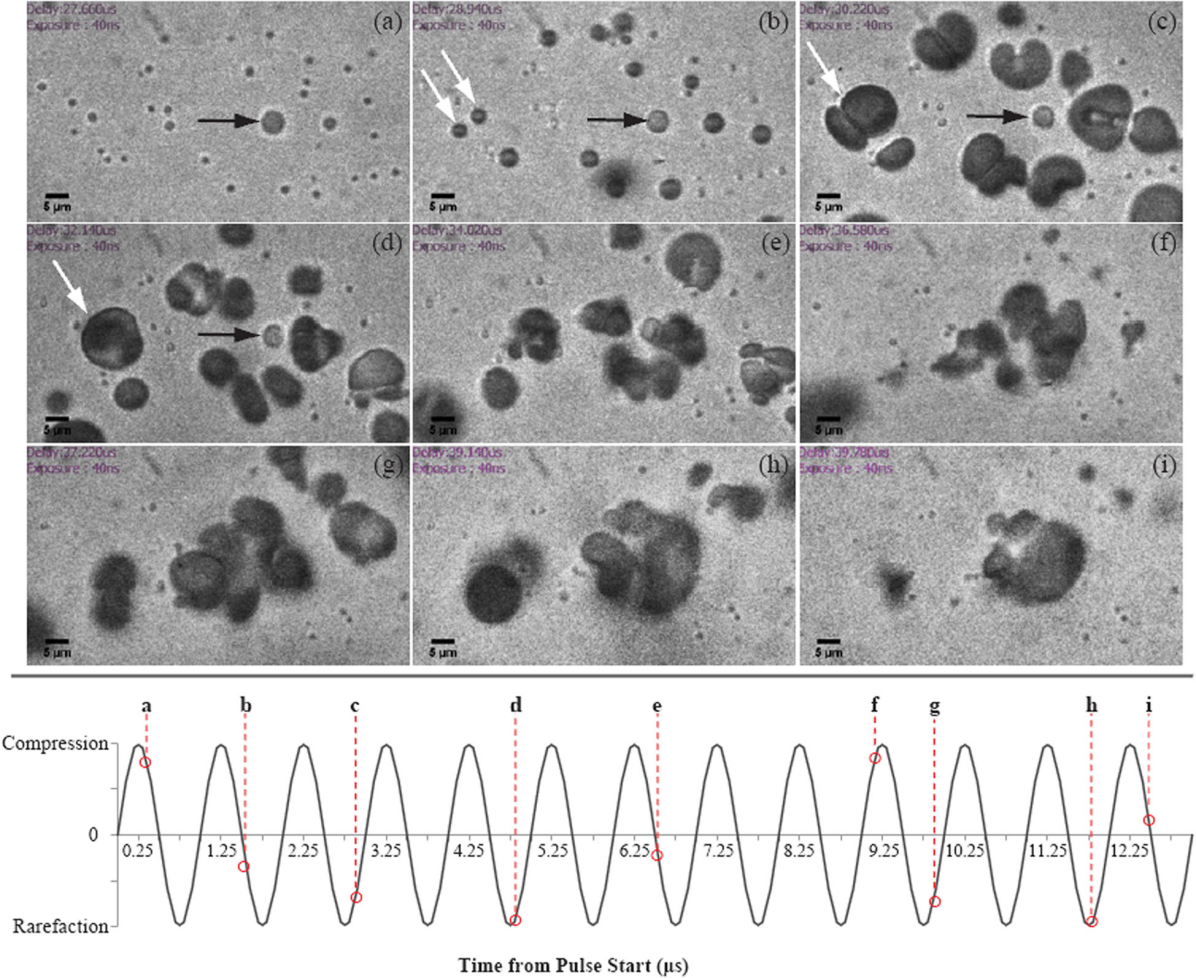
Shadowgraphs of bubbles dynamics using a 20-cycle 1 MHz pulse of 1.45 MPa rarefactional pressure show the droplet vaporization and fully expansion within 1–2 μs (**a**–**d**), microbubble fusion (**b**–**d**) (*white arrows*), and radiation force (**d**–**i**), scale bars are 5 μm, with courtesy of [34]

Bubbles vaporized from droplets are significantly affected by subsequent ultrasound pulses. These secondary effects, such as microbubble destruction, coalescence fusion, and acoustic radiation force, increase significantly with acoustic pressure and pulse length interactions. Therefore, in order to minimally affect the generated microbubbles, ultrasound pulses with relatively short duration should be applied to initiate vaporization. Each positive pressure phase may destroy some bubbles produced in the prior rarefactional phase, but not completely. Thus, many previously visualized bubbles re-emerge in the sequential rarefactional trough (see Fig. 6). Small bubbles are more likely to be destroyed by compressive pressure, while the large ones have resistance to destruction and high possibility of bubble fusion [34].

The performance of droplets in the acoustic field is determined by many parameters, such as ultrasound pulses, droplet properties, and environmental variables. A viscous fluid, limited tissue space, and high elasticity of surrounding tissue may retard the initial expansion of the gas bubble generated by ADV and re-condense the gas nucleus at lower rarefactional pressures. Greater acoustic amplitude will start bubble growth sooner and have more total time for growth. Similarly, low-frequency ultrasound provides a longer time window for growth. For example, using a 2-cycle sinusoid pulse at the ultrasound frequency of 1 MHz, bubble formation was found at rarefactional pressure threshold of 1 MPa, while at 8 MHz the threshold reached approximately 3 MPa for decafluorobutane nanoemulsions with peak diameter of 200 nm [34]. The ADV threshold was shown to be inversely proportional with degree of superheating and independent of pulse length at clinically relevant ultrasound frequencies, concentrations of droplet, and the volume fractions of the PFC, which may be useful for in vivo application because of limited information of droplets on site [25, 35, 36]. For longer pulse lengths (i.e., milliseconds), vaporization can be induced at a decreased ultrasound pressure. In comparison, for microsized droplets (90 % < 6 μm), the vaporization drops with the ultrasound frequency, from 4.5 to 0.75 MPa peak rarefactional pressure between 1.5 and 8 MHz [8]. It takes a longer time for the microdroplets to completely vaporize than the submicron ones. However, a larger emulsion grows into a larger bubble, which is due to a longer time of subpressurization and a larger subpressurization driving force by the smaller Laplace pressure and the initiation of gas evolution earlier in the acoustic cycle.

## Manufacture

Perfluorocarbon (PFC) including perfluorohexane (C_6_F_14_), dodecafluoropentane (DDFP, C_5_F_12_), and decafluorobutane (DFB, C_4_F_10_) is a common material for liquid emulsion formulation. The use of others, such as phosphatidylethanolamine (PE), soybean phosphatidylcholine (PC), pefluoropentane (PFP), perfluorohexane (PFH), perfluorobutane (PFB), perfluoro-15-crown-5-ether (PFCE), perfluorooctylbromide (PFOB, C_8_F_17_Br), and perfluorotripropylamine was also mentioned [21, 37–40]. Some manufacture approaches are listed below.

The first method is similar to that of microbubble manufacture. Liquefied PFC gas and degassed, deionized water were mixed and emulsified with an ultrasonic liquid processor for about 30 s. Sometimes, coarse emulsification by a vortex mixer or an amalgamator was performed before the use of sonicator. The resulting emulsion was poured slowly into albumin or lipid (i.e., phospholipids) or polymer solution or surfactant to coat the droplet, and the shell was used to stabilize the emulsion by lowering the surface tension as well as inhibiting coalescence. These uncoated emulsions are stable for 30–60 min and then start to coalesce after 2–3 h, which may be due to the nature of hydrocarbon. The surfactant (i.e., PF68) can interact with phospholipid shells and increase the surface area of each droplet [41]. Subsequently, the size of the emulsions decreases with higher surfactant ratio. But, drug release from the inner core may be much slower than that from the interfacial shell [42]. Double emulsion (i.e., oil-in-PFC-in-water) could also be manufactured using similar method [24]. In addition, micelles and liposomes could also be produced depending on the lipid concentration. This method is simple in manufacture. In order to have uniform size distribution, the emulsion needs to pass through filter paper with certain pore size, which may reduce the manufacturing efficiency.

Droplet could be formulated via extrusion. PFC was first condensed in a secure container over dry ice, poured into a glass vial, crimped, and stored at −20 °C to preserve the liquid state. The lipid solution was cooled to approximately −2 to −5 °C in order to avoid freezing of the aqueous solution and then mixed with liquid PFC. The mixture was extruded by several passes through porous membrane filter. After that, the emulsion was stored in a crimped vial at 4 °C with room air in the headspace [43]. Both the submicrometer and the micrometer size (as large as 12–15 μm) droplets were made via extrusion.

Microbubble condensation method induced by pressurization and low temperature allows simple production of high-yield nanoemulsions from volatile compounds [43]. Polydisperse distribution of microbubbles was first produced using standard mechanical agitator, cooled to room temperature before being immersed in a CO_2_/isopropanol bath at −5 to −10 °C, and swirled gently for about 1 min. Headspace pressure in the vial was then increased by an adjustable air-pressure source to revert the emulsions to the liquid state. Pressurization and temperature condensation of microbubbles are effective and advantageous in producing ADV nanoemulsions in comparison to extrusion and emulsion-based methods. The condensed droplet size has a peak of 200–300 nm, which corresponding well with the prediction using the ideal gas law from the original bubbles in the 1–2 μm range. Variability in the droplet size (some samples having content below 100 nm while others having only content greater than 200 nm) may be due to both insistencies in applied pressure and temperature at the time of condensation and formation of micelles and liposomes as a function of lipid concentration. Some content as large as 2–4 μm was also found in the sample despite of low percentage (1.5–4 %), and the upper size limit seems to increase with lipid concentration. The samples with a high number of viable outputs could be obtained using a similar technique as traditional microbubble preparation [43].

A microfluidic channel with a flow-focusing structure can also be used for uniform nanoemulsion production by forcing a central stream of a dispersed phase and two side sheath flows of a continuous phase through a small orifice. The size of droplets including the shell thickness is well controlled and adjusted by the geometry of microfluidic channel and flow rate. The minimum size is the width of the orifice. A high capillary number will lead to droplet generation in the jetting mode, and breaking off the tip of the dispersed phase finger due to Rayleigh capillary instability will result in polydisperse production with a polydispersity index less than 5 % [44]. In addition, over 2 weeks, the mean droplet diameter decreased less than 4 % from 4.5 ± 0.2 μm to 4.3 ± 0.3 μm. However, manufacture speed of this method is not very high. Multiplexing numerous flow-focusing circuits would scale the throughput. A 10× scale-up would significantly reduce the production time of 1 × 10^9^ droplets on the order of minutes similar to the amount and time formed by mechanical agitation.

## Applications

### Vessel occlusion (embolotherapy)

It is suggested to “starve” cells to death by restricting their blood supply from the feeder vessel [45]. One method of treating tumors or other malformations is to occlude the blood flow to them with gas bubbles, which can effectively shrink its size. By using focused ultrasound, vaporization can be induced intentionally and accurately in the feeder arteries of tumors, resulting in the occlusion with a high degree of spatial specificity [15, 16]. For frequencies between 1.5 and 8 MHz, the threshold of peak rarefactional pressure for vaporization decreases from 4.5 to 0.75 MPa for microdroplets (90 % < 6-μm diameter) [8]. Large gas bubbles (>30 μm) could be formed temporarily. Vessel occlusion via ADV has been explored in rodents and dogs (76-μm mean length and 36-μm mean diameter in capillary and 25-μm mean length and 11-μm mean diameter in feeder vessel) and may be translated to clinical use soon [16]. Image-based hyper-echogenicity from ADV of intra-articular (IA) and intravenous (IV) injections after sonic exposure (9.2-MPa peak negative pressure, 3.5-MHz frequency, 13 cycles, pulse repetition frequency of 1 kHz and I_SPTA_ of 10 W/cm^2^) was monitored for approximately 90 min, and cortex perfusion was reduced by >60 % of its original value for more than 1 h, which could be long enough for the onset of cell death and possible tumor treatment via ischemic necrosis. However, in these studies, the control kidney on the contralateral side also showed 18 % of the decrease in regional blood flow relative to the preocclusion baseline, which may be due to the balance of the urinary output between the treated and untreated kidneys [15]. IV administration results in a lower gas bubble yield, which may be due to the filtering in the lung, dilution in the blood volume, or other circulatory effects. So, large volume of the droplet is required for IV administration in comparison to IA injection (0.3 vs. 0.03 mL) [46]. However, a raft of recent data shows that starving of a tumor can lead to increased epithelial-to-mesenchymal transition and metastatic escape leading to worse overall survival in patients [47, 48]. Thus, the future of this approach is unclear.

In comparison to the other vascular occlusion modalities, ADV could improve both diagnostic and therapeutic ultrasound fields. ADV-based angiography can provide sensitive feedback on the effect of ultrasonic therapy in models of pancreatic cancer, breast cancer, and kidney function. Despite clinical competition from other modes of vascular occlusion, ADV-induced embolotherapy has great potential in vital organ tissues (brain, liver, eye, etc.), in which revascularization after therapeutic healing is desired. Occlusion can also be accompanied by deposition of a chemotherapy drug to increase local cytotoxic effects and minimize systemic effects [15]. For example, thrombin release from PFC emulsion could extend the duration of ADV-generated microbubble occlusions. Angiostatin and endostatin suppress the development of supply arteries for tumor growth [8].

ADV-generated occlusion also has the potential to instigate hemostasis for vascular damage or internal bleeding. Vaporizing in the capillaries could cause the rupture of these vessels and subsequent red blood cell extravasation. The encapsulated chemical embolic agent, such as thrombin, within a PFC emulsion could be released locally and noninvasively upon ADV with precision on the order of millimeters and with no need for ionizing fluoroscopy as in transcatheter embolization for sustained embolization. Furthermore, prolonged ischemia generated by vascular occlusion may activate water soluble, bioreductive prodrugs, such as NLCQ-1 [49], encapsulated within the emulsion [50].

Occlusion is also beneficial in the thermal ablation induced by radio frequency, microwave, or high-intensity focused ultrasound (HIFU). The blood flow in the tumor acts as a heat sink, dissipates the heat via vascular cooling, and subsequently reduces the efficacy of the treatment. Reduced blood flow by occlusion could also induce hypoxia in tumors, but such occlusion prevents drugs diffusion into the target.

Overall, embolotherapy must be carried out carefully because many arterial emboli could create infarcts in the heart or brain or travel to distant vascular bed where they could cause unwanted arterial occlusion, ischemia, and potentially infarction [51]. The performance of ADV-based occlusion is determined by the dynamics of emulsion and bubbles in the transport (i.e., interaction with vessel bifurcations downstream) and lodging of the generated microbubbles in the microvasculature near the site of vaporization (i.e., sliding along the vascular space). If the droplets have stealth character, the generated gas bubbles will easily coalesce into sufficiently large one(s) to occlude arterioles and capillaries. However, vascular occlusion may not work well for superficial tumors. It was found that reduction in blood flow lowers the core temperature of superficial tumors, which in turn increases the survival of tumor cells by approximately two orders of magnitude [52, 53]. Moreover, significant growth delays were found to require occlusion times on the order of 4 h, which requires repetitive treatments of ADV.

### HIFU ablation

**Fig. 7:**
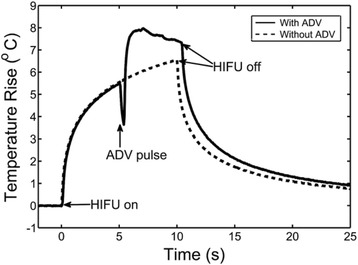
Temperature elevations measured during a 10-s continuous HIFU exposure (2 MHz), and the ADV pulse (2 MHz, 5 cycle, 7.05 MPa peak negative pressure) was fired at 5 s, which results in a short temperature drop, and then back to continuous mode for heat deposition, with courtesy of [19]

Because of the much lower acoustic impedance of gaseous bubbles in comparison to the surrounding soft tissue, transformation of ultrasonic pressure waves to thermal energy is much more efficient in the presence of microbubbles, producing localized viscous heating. ADV may significantly and locally enhance thermal delivery in the focal region from phase-shift nanoemulsions once vaporized for cancer therapy that hopefully will soon be demonstrated in a preclinical setting. A high degree of spatial and temporal control of emulsion vaporization could also be beneficial in generating predictable lesions. Once the ADV pulse was transmitted and the DDFP droplets were vaporized after only a 5-cycle exposure at high pressure, a sudden and dramatic rise in temperature was recorded at continuous HIFU exposure at much lower pressure, which is characteristic of bubble enhanced heating (see Fig. 7). It was found that the exposure time needed to create similar lesions in tissue-mimicking phantoms filled with droplets was decreased by a factor of 2.5 and the average lesion volume increased by sevenfold using the equal exposure times. In vivo results in the canine liver were even more promising, showing a 15-fold increase in lesion volume at equal exposure time to controls without droplets [10].

In the acoustic field, bubble shielding effects are of importance, in which the resident bubbles will reflect or back scatter the energy of incoming acoustic pulses toward the source. As a result, it is possible to vaporize additional nanoemulsions easily in the prefocal region during HIFU tumor ablation, which could lead to unpredictable prefocal lesion formation [19]. In HIFU ablation, PFCs with a higher boiling point, such as PFH, may be more appropriate. Inertial cavitation may significantly reduce the ADV threshold for HIFU exposures longer than a millisecond. Vaporizing albumin-coated DDFP microdroplets by heat alone required temperatures as much as 40 °C above the boiling point [35], and such discrepancy may increase as the droplet size decreases. In addition, vaporized microbubbles may be manipulated to enhance targeting through acoustic radiation force [54–56].

### Drug delivery

Targeted tumor chemotherapy is an active research area with great potential. The concept of a “magic bullet,” a drug carrier that responds to a certain stimulus, was first proposed by Ehrlich in the early twentieth century. However, most drug delivery techniques do not have the capability of temporally and spatially specific targeting as ultrasound. The control of localized delivery is especially important for drugs which possess narrow therapeutic windows and will allow the deleterious effects on healthy tissues to be minimized. Ultrasound also provides the ability of target diagnosis and focusing onto deeply located tissues. Drug-loaded microbubbles under ultrasound exposure can potentially target drugs to specific sites. Therapeutic agents are typically incorporated into the microbubbles by attachment to or insertion in the shell, complexation of secondary carriers to the microbubble shell, or incorporation within a fluid inside the shell. Chemotherapeutic drug (i.e., paclitaxel, PTX) was tightly retained by nanodroplets stabilized with poly(ethylene oxide)-co-polycaprolactone (PEG-PCL) block copolymer. But, it was effectively released into tumor after acoustic radiation, which resulted in effective tumor regression [38, 57]. The interaction of ultrasonic pulse with a payload containing microbubbles involves a number of mechanisms, such as acoustic cavitation, heating, radiation forces, and sonoporation [58]. Stable bubble cavitation generates strong shear stress close to the bubble surface, sufficient to shear cell membranes. Inertial cavitation produces shock waves and high-speed microjets, which also disrupt cell membranes. The transient increase in cell membrane permeability allows the uptake of drugs, genes, and peptides from a variety of carriers (polymeric micelles, liposomes, and nanoemulsions).

**Fig. 8:**
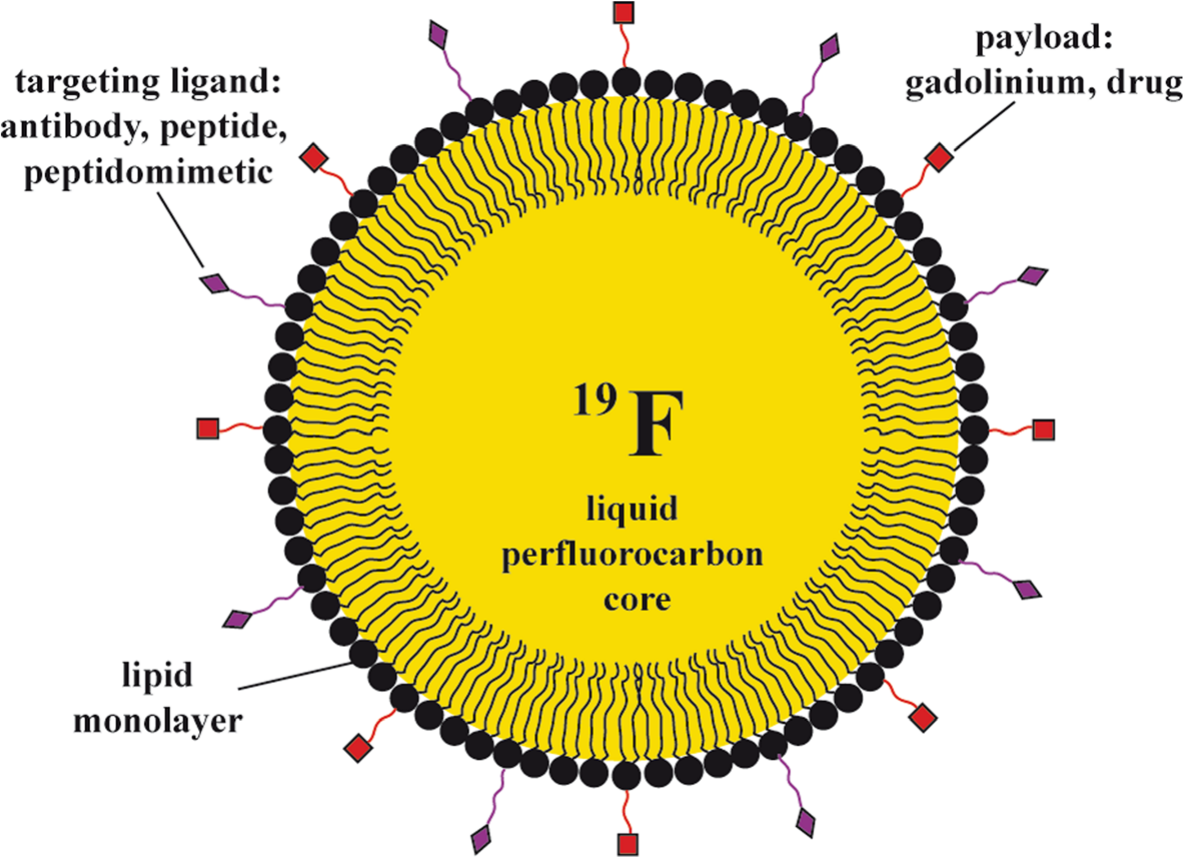
Schematic diagram of a liquid perfluorocarbon nanoemulsion with targeting ligands and payload

Ultrasound can also be used to spatially and temporally control the release of a therapeutic payload (i.e., water-soluble compounds) encapsulated within a PFC nanoemulsion via ADV. Since PFCs are very hydrophobic and lipophobic [59], therapeutic agents are usually not dissolved directly in the PFC phase but rather in a secondary phase that is contained within the PFC emulsion [60]. The PFC droplet, either in the liquid or gas state, works as a contrast agent to visualize and confirm the location of the desired delivery. Higher-intensity ultrasound is delivered to generate strong cavitational events to disrupt carriers or cell membranes only if the location is correct. Because of the absence of endogenous background signal in vivo and the high nuclear magnetic resonance (NMR) sensitivity of the ^19^F atom, PFC nanoemulsions are ideal agents for cellular and magnetic resonance molecular imaging. Due to the hydrophobicity and lipophobicity of the dispersed PFC phase [59], therapeutic agents are usually incorporated into the emulsion. The PFC core is surrounded by a lipid monolayer which contains a variety of agents including targeting ligands, imaging agents, and drugs either individually or in combination. One example is shown in Fig. 8 [50]. Plenty of targeting ligands (~20–40 monoclonal antibodies or 200–400 small-molecule ligands) serve to enhance avidity through multivalent interactions while the PFC core has high local concentrations of ^19^F [61, 62]. Fluorine atoms have the unique property of being both lipophobic and hydrophobic. The PFC nanodroplets were stabilized by some of the block copolymer and the micelles, with the drug distributed in both the micelles and the nanodroplet surface.

**Fig. 9:**
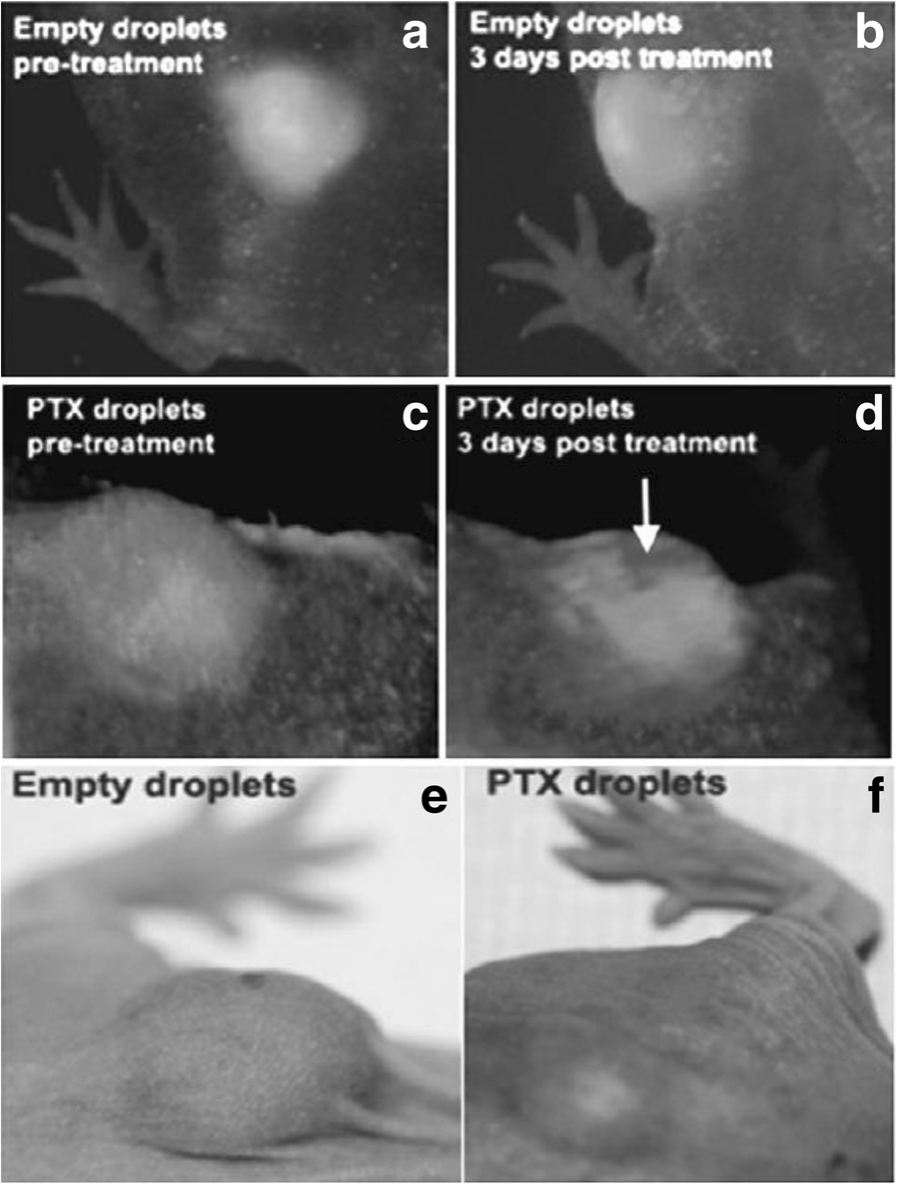
Intravital fluorescence images of subcutaneous pancreatic tumors in mice injected with empty droplets (**a** and **b**) or PTX-loaded 1 % PFCE/5 % PEG-PDLA droplets (**c** and **d**) 6 h before and three days after ultrasound therapy (50 s exposure in a circle of 4-mm diameter, 1 MHz frequency, acoustic intensity of 54 W/cm^2^), and photographs of the tumors taken 12 days after the treatment (**e** and **f**), with courtesy of [64]

**Fig. 10:**
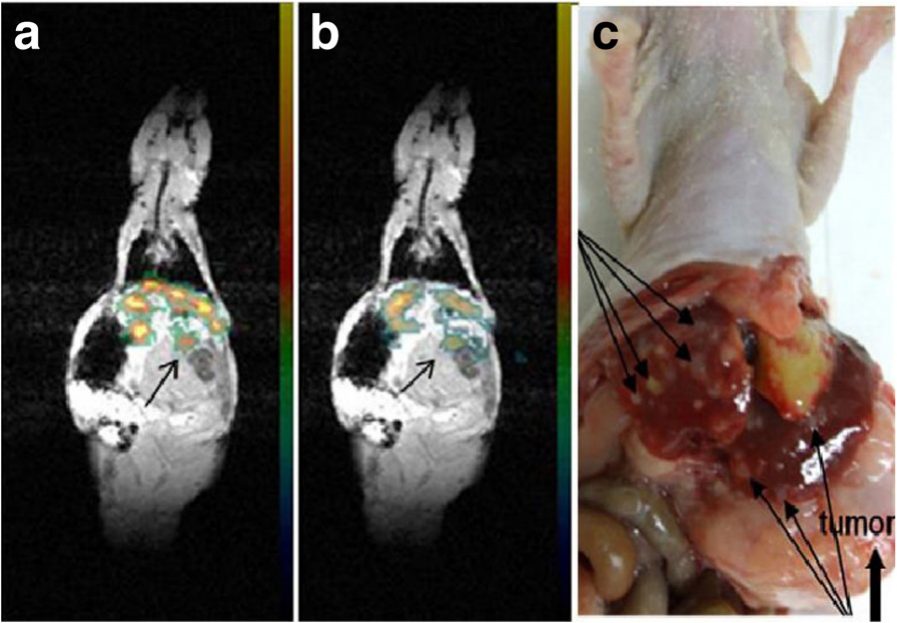
A 2 % PFCE nanoemulsion stabilized with PEG-PCL copolymer was systemically injected to a pancreatic tumor bearing mouse every 2 h 200 μl each for four times and a total PFCE dose of 2 mmol/kg. Coronal slices in the ^19^F MR images superimposed on the low resolution proton anatomic images (**a** and **b**) were recorded an hour after the last nanodroplet injection (7 h after the start). Multiple liver metastases (*long thin arrows*) and transparent large tumor (*thick arrow*) are revealed at the necropsy of the mouse (**c**), with courtesy of [64]

ADV for drug delivery is still in preclinical stages with initial applications to treat tumors in mice and retard tumor growth. Nanodroplets may be able to extravasate and become acoustically activated or form gas bubbles, and cavitation may have occurred in the capillaries in the tumor to increase capillary permeability for drug delivery. Systemic doses of anti-angiogenic compounds (TNP-470) have been lowered by 1000-fold and 60-fold in the animal and clinical studies using the targeting techniques, respectively [63]. Ultrasound therapy (2 MPa) using paclitaxel-loaded PFCE nanoemulsions in the breast and pancreatic cancer animal showed excellent tumor regression and metastasis suppression as shown in Figs. 9 and 10 [64]. In addition to the mechanical permeabilization of cell membranes (sonoporation), acoustic radiation force and microstreaming may push nanoparticles through blood capillary walls to enhance the drug extravasation, and a temperature increase may also lead to cell membrane fluidization [65].

**Fig. 11:**
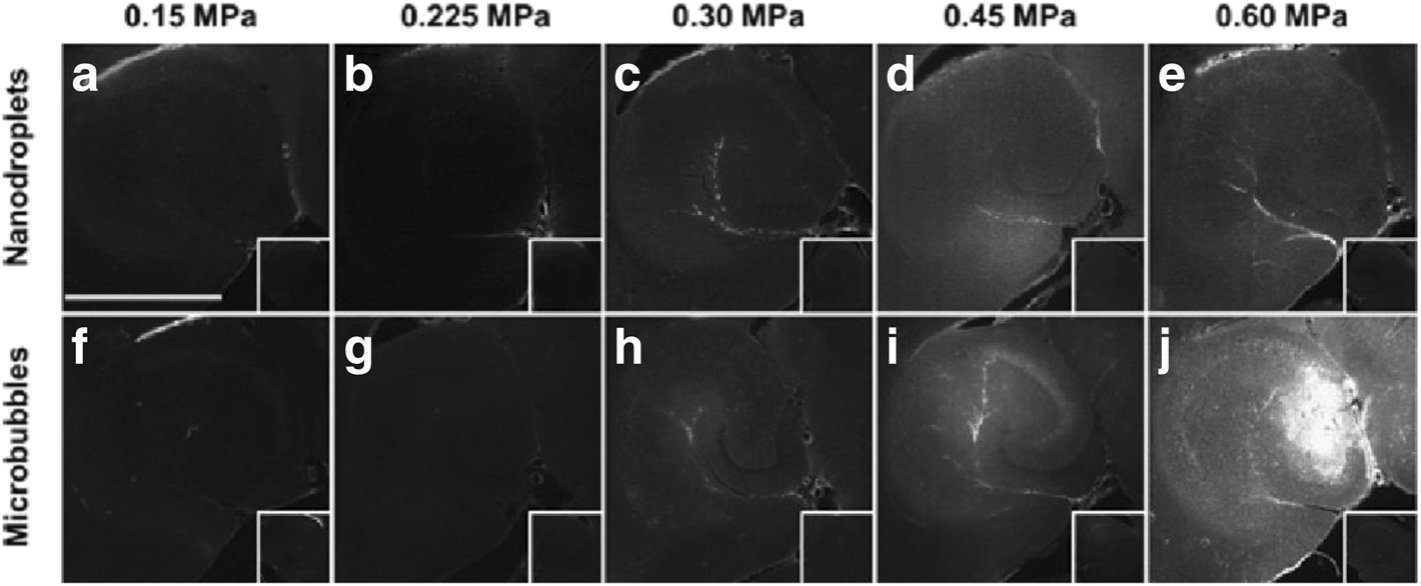
Representative fluorescence images in the targeted and the control (*insets*) hippocampi when nanodroplets (*top*) or microbubbles (*bottom*) and fluorescently labeled 3 kDa dextran were used to mediate BBB opening at frequency of 1.5 MHz, pulse duration of 6.7 ms, pulse repletion frequency of 5 Hz, exposure duration of 5 min, various peak-rarefactional pressures of 0.15 MPa (**a** and **f**), 0.225 MPa (**b** and **g**), 0.30 MPa (**c** and **h**), 0.45 MPa (**d** and **i**), and 0.60 MPa (**e** and **j**), the scale bar is 1 mm, with courtesy of [27]

Cavitation activities of UCAs under the exposure of HIFU pulses have already been illustrated to noninvasively and effectively cause the reversible blood-brain barrier (BBB) opening after a 5-s sonication (pulse length of 6.7 ms, pulse repetition frequency of 5 Hz, acoustic pressure of 0.6 MPa as shown in Fig. 11) [66, 67]. Shear stress-induced endocytosis during stable cavitation is one of the important mechanisms of increasing the transcellular permeability [68]. The cavitation dose can be calculated from the measured acoustic emission signals and then correlated with the outcome of the BBB opening to estimate the amount of drug delivered to the targeted region. Similar to drug delivery in the tumor, once entered into the interstitial cerebral space, the droplets could then be vaporized acoustically activated to form microbubbles outside the constraints of the cerebral microvessels as a new class of contrast agents. Such extravascularly activated microbubbles can enhance drug delivery at deeply located sites in the brain tissue or in regions with relatively low vasculature density [27]. Stable cavitation dose increases in a linear relationship with the acoustic pressure for both nanodroplet (*R*^*2*^ = 0.99) and microbubbles (*R*^*2*^ = 0.93). There was no detectable inertial cavitation dose at the pressure up to 0.6 MPa, suggesting no bubble fragmentation. However, the inertial cavitation dose slightly increased after microbubble injection at 0.60 MPa (*P* = 0.017). Overall, nanodroplets had a higher pressure threshold for BBB opening but a lower stable cavitation threshold than the microbubbles. Although similar homogenous dextran distribution throughout the targeted volume was achieved using both vaporized nanodroplet and microbubbles under the same acoustic exposure settings, microbubble-mediated BBB opening resulted in minor hippocampus damage at 0.60 MPa. Therefore, a higher acoustic pressure could be applied to nanodroplets safely because of its higher threshold for inducing inertial cavitation than that of microbubbles. These two agents have the same composition, lipid-encapsulated perfluorobutane cores, but the nanodroplet has both the liquid and gaseous states of the cores. The large microbubbles formed by nanodroplets vaporization may enhance the shear stress induced during stable cavitation to endothelial cells along the microvessels more efficiently than UCA microbubbles, thus increasing the number of BBB disrupted sites to facilitate a more homogenous dextran delivery distribution without concerns regarding undesired tissue injury due to inertial cavitation.

### Histotripsy

**Fig. 12:**
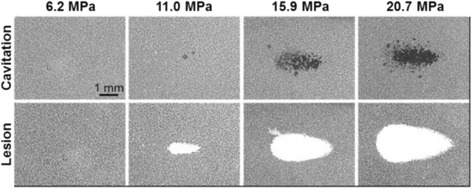
Optical images of nanodroplet-mediated cell fractionation. Images of cavitation bubble cloud (*dark*) and lesions (*white*) generated in the RBC agarose gel (*gray*) using nanodroplet-mediated histotripsy at different pressure levels. A total of 2000 2-cycle pulses at a driving frequency of 500 kHz and a pulse repetition frequency of 10 Hz were used for each treatment with peak negative pressure from 6.2 to 20.7 MPa, with courtesy of [77]

Using extremely short but high-pressure pulses, histotripsy generates a dense cloud of cavitation microbubbles that fractionates tissue. Synthetic nanodroplets that encapsulate a PFP core will transition upon exposure to ultrasound pulses into gas microbubbles, which will rapidly expand and collapse resulting in disruption of cells similar to the histotripsy process but at a significantly lower acoustic pressure. The significantly reduced cavitation threshold will allow histotripsy to be selectively delivered to the tumor tissue and greatly enhance the treatment efficiency while sparing neighboring healthy tissue. A bubble cloud occurs after the first pulse, and then the number of bubbles inside the cloud significantly decreased with increasing number of pulses and finally will be destroyed by the cavitation process, which is more pronounced at higher frequencies. Nanodroplet-mediated histotripsy (NMH) created consistently well-defined fractionation of the cells in agarose tissue phantom similar to the lesions generated by histotripsy alone but at a significantly lower pressure (see Fig. 12). Once the cavitation bubble cloud is initiated, the histotripsy process can be maintained at a much less pressure than that needed for initiation. The resulting gas bubbles can function as ultrasound contrast agents, which will allow the histotripsy to be seen on sonography for real-time guidance and monitoring. The energetic bubble activity disrupts adjacent cells and eventually produces complete fractionation of cells in the target tissue into a liquefied homogenate. A peak negative pressure below 10 MPa, no microbubbles were observed and no lesions were visualized. In comparison, in control gels without nanodroplets, no lesions were formed at any treatments in this pressure range. Lower frequency would improve the effectiveness of NMH by increasing the size of the focal region, increasing bubble expansion, the number of bubbles generated, and the lifespan of bubble cloud which is due to the enhanced bubble expansion results in a larger population of residual nuclei and a corresponding increase in dissolution time. No bubbles were observed after 100 pulses delivered to the samples. NMH will be applied at a higher pulse repetition frequency to sustain cavitation for the duration of the treatment. Nanodroplet containing a higher boiling point perfluorocarbon, such as perfluorohexane, would re-condense into a liquid and remain sustainable nuclei over multiple pulses, which may be a major benefit for NMH therapy. However, higher boiling point droplets may also require a higher pressure to generate cavitation [69].

## Discussion

In the past decades, extensive studies have been carried out to understand the mechanism of ADV, simulate the bubble dynamics in the acoustic field, and apply this phenomenon in medical investigation. Due to the complexity involved in this event, including nucleation, vaporization, oscillation, recondensation, and dissolution, and a large range of droplet size used in the study (both submicron and micron), more effort is required to fully understand the mechanisms. Some insights have already been obtained owing to the use of ultrafast photography with a large number of frames. Meanwhile, in order to enhance its clinical performance and safety for quick transition and wide acceptance of the applications, some aspects, but not limited, may have more interests for continuous investigation in the following days.

Inclusion of paramagnetic nanoparticles, fluorescent nanoparticles, and radioisotopes enables the detection of PFC emulsions in ^1^H MR T1-weighted imaging, SPECT-CT, and optical fluorescence imaging [70–73]. Fluorine NMR has a large chemical shift range (~300 ppm) allowing the examination of multiple agents simultaneously with minimal signal overlap. The integral of quantitative NMR signal, such as the fluorine atoms within the core of PFC nanodroplets, is proportional to the amount of particles being interrogated for MR molecular imaging. The ^19^F isotope of fluorine has a natural abundance of near 100 %, but virtually zero in the biological presence. Therefore, the chemical shift of the PFCs can be easily differentiated from the background. The nanoparticles for molecular imaging must have a long circulation time, highly sensitive and selective binding to the epitope of interest, prominent contrast-to-noise enhancement, acceptable toxicity, ease of clinical use, and compatibility with commercially available imaging systems. Multifunctional activity can be realized by incorporating more targeting ligands, imaging agents, and drugs into the formulation simultaneously. Materials can be covalently or noncovalently linked to the particle surface, dissolved in the coating, or carried in particle interiors for cellular deposition and activation. Multivalent interactions between high-avidity agents and cell surface may partially overcome the dissociation of the targeting ligand itself in the nanomolar range [10].

Most of the ADV techniques mentioned above are still in preclinical studies but have potential for clinical use in specialty applications. Overall, ADV has a bright future because the small size of nanodroplets greatly reduces the rate of clearance compared to larger ultrasound contrast agent bubbles and yet provides the advantages of ultrasonographic contrast, acoustic cavitation, and acceptable toxicity of conventional perfluorocarbon contrast agent bubbles. Injection of fluorocarbon emulsions induces short- and long-term effects that spontaneously resolve within 12–24 h. The adverse effect of “pulmonary hyperinflation” observed upon administration to humans is less likely than that for rabbits, pigs, or monkeys. In addition, toxicity of surfactants that stabilize the droplets must also be considered. Natural phospholipids, polysaccharides, and human proteins may be the best stabilizing agents to use.

The capability of simultaneous imaging and targeted drug delivery of nanoparticles shows great promise for individualizing therapeutics and could enable conclusive assurance that the drug is reaching the intended target in a much higher effective drug concentration. Imaging can also be used to monitor distribution of released drugs. For example, liposome colabeled with gadolinium (Gd) could allow MRI monitoring. Fluorescent markers on the drug can be detected using bioluminescent imaging system. Such information allows optimal timing of external stimulus application. Furthermore, approval by regulatory agencies may be very slow although the FDA has specific pathways for combined devices that go through two separate departments [74]. This regulatory obstacle may temper the enthusiasm of pharmaceutical companies to pursue development, given the expense and risk of clinical trials [10]. ADV is a complex process and not a fully understood phenomenon, involving many variables that are not always straightforward to regulate for safety and may be some variables not taken into consideration yet.

New treatment strategies will be developed, and operation parameters will be optimized in the following years to enhance the outcome. The internalization of targeted or nontargeted droplets into cells and subsequent vaporization could lead to new tissue-specific therapy. A co-injection of both nano- and microdroplets could simultaneously release drugs in the tumor interstitium by extravasated nanodroplets and occlude the vascular space by larger ones [10]. If the lowest vaporization pressure is required, the PFC with the lowest boiling point allows for stable circulation at physiological temperatures will be used. The optimal particle size of the emulsion should result in a vascular persistence that is clinically efficacious while limiting deep-tissue retention. Droplets may remain stable until a desired “activation pulse” is delivered to create desired bioeffects. More measurements are required to establish the acoustic thresholds for gas expansion as a function of acoustic parameters (frequency, amplitude, pulse length, etc.) and the characteristics of the droplets (chemical composition, size, stabilizing surfactants, temperature, etc.) [75].

## Conclusions

Acoustic droplet vaporization, a phenomenon of phase shift from liquid to gas in the acoustic field, is a new approach of producing bubbles to enhance the bioeffects of ultrasound therapy with high accuracy of spatial and temporal control. The burst produced from commercially available echosonographic systems is strong enough to induce ADV for microdroplets. Submicron size of droplets allows penetration into tumors via EPR effects and much prolonged half-life time in the circulating system, overcoming the limitation of the commonly used ultrasound contrast agents (i.e., microbubbles), but needs more acoustic pressure to vaporize. Its recent applications, such as vessel occlusion, thermal ablation, drug delivery, and histotripsy, have already shown very promising results. Although most in vivo studies are still in the preclinical stage, translation into clinics may occur in the following years. In comparison to bubble cavitation of microbubbles, that of vaporized droplets has more stable cavitation and less inertial cavitation, which makes ADV good candidate for localized drug delivery with the minimal damage to the surrounding tissue. In order to understand the mechanism more clearly, effort in the theoretical modeling and experimental observation is highly desired.
